# Predicting native papilla biliary cannulation success using a multinational Endoscopic Retrograde Cholangiopancreatography (ERCP) Quality Network

**DOI:** 10.1186/1471-230X-13-147

**Published:** 2013-10-10

**Authors:** Chunyan Peng, Paul J Nietert, Peter B Cotton, Daniel T Lackland, Joseph Romagnuolo

**Affiliations:** 1 Division of Gastroenterology and Hepatology, Medical University of South Carolina, 25 Courtenay Drive, ART 7100A, Charleston, SC 29425, USA; 2 Division of Gastroenterology, the Affiliated Drum Tower Hospital of Nanjing University Medical School, Nanjing, P R. China; 3 Department of Public Health Sciences, Medical University of South Carolina, Charleston, SC USA; 4 Department of Neurosciences, Medical University of South Carolina, Charleston, SC USA

## Abstract

**Background:**

Success in deep biliary cannulation via native ampullae of Vater is an accepted measure of competence in ERCP training and practice, yet prior studies focused on predicting adverse events alone, rather than success. Our aim is to determine factors associated with deep biliary cannulation success, with/ without precut sphincterotomy.

**Methods:**

The ERCP Quality Network is a unique prospective database of over 10,000 procedures by over 80 endoscopists over several countries. After data cleaning, and eliminating previously stented or cut papillae, two multilevel fixed effect multivariate models were used to control for clustering within physicians, to predict biliary cannulation success, with and without allowing “precut” to assist an initially failed cannulation.

**Results:**

13018 ERCPs were performed by 85 endoscopists (March 2007 - May 2011). Conventional (without precut) and overall cannulation rates were 89.8% and 95.6%, respectively. Precut was performed in 876 (6.7%). Conventional success was more likely in outpatients (OR 1.21), but less likely in complex contexts (OR 0.59), sicker patients (ASA grade (II, III/V: OR 0.81, 0.77)), teaching cases (OR 0.53), and certain indications (strictures, active pancreatitis). Overall cannulation success (some precut-assisted) was more likely with higher volume endoscopists (> 239/year: OR 2.79), more efficient fluoroscopy practices (OR 1.72), and lower with moderate (versus deeper) sedation (OR 0.67).

**Conclusion:**

Biliary cannulation success appears influenced by both patient and practitioner factors. Patient- and case-specific factors have greater impact on conventional (precut-free) cannulation success, but volume influences ultimate success; both may be used to select appropriate cases and can help guide credentialing policies.

## Background

Predicting quality is an important part of determining training, credentialing, and recredentialing thresholds. Endoscopic retrograde cholangiopancreatography (ERCP) is widely performed to diagnose and treat pancreatic and biliary disorders, at 1 per 1000 population, estimated at 3–500,000 annually in the US. Post-ERCP pancreatitis is the most common adverse event (1% to 7%, up to 10-20% in high risk patients) [1-4], accounts for most of the related mortality (0.1%), an estimated 500 deaths/year in the US [5,6]. Pancreatitis is more likely after repeatedly failed cannulation [7], thus maximizing cannulation success is important, not only for avoiding costly repeat and rescue procedures, but also for decreasing adverse events.

In community practice, ERCP is most often performed for biliary diseases [8], with the rate of successful biliary cannulation remaining the key performance metric. Minimum standards of 80-90% have been proposed internationally [9,10], yet wide practice variations (54%-98%) exist. This rate variation can only be partly explained by variable exclusions of precut sphincterotomy, and cut/stented papillae [11-14]. Although “pre-cut” sphincterotomy to facilitate access in difficult cases may increase risk in inexperienced hands, recent meta-analyses of randomized trials concluded that precut appears safer than persistence for experienced endoscopists [15]; and may arguably no longer be regarded as “failure”, at least as a secondary outcome. It would also seem preferable to exclude previously stented/cut papillae.

Volumes, training, and practice conditions may play a role [16,17], yet these factors have not consistently been strong predictors [8]. Difficulty of the procedure may also contribute [18,19]. The influence of other factors (trainees, comorbidity, sedation) remains largely unknown. Therefore, data gathered in a unique multinational ERCP Quality Network, were used to investigate the predictors for native papilla biliary cannulation success (with and without “precut”) using multilevel logistic regression analyses.

## Methods

### Data sources and study cohort

The data were retrieved from the ERCP Quality Network database, a web-based registry of prospectively entered, consecutive, self-reported, anonymous data from a variety of ERCP practices worldwide (March 28, 2007 - May 18, 2011). Data was cleaned, excluding cut/stented papillae, physicians contributing <30 cases, and cases without biliary cannulation attempts. Informed consent was waived by the Medical University of South Carolina Institutional Review Board, and the study of this database without patient identifiers was granted an exempt status.

### Outcomes and study variables

The primary outcome was “conventional” deep biliary cannulation success, with use of precut considered a “failure”. The secondary outcome was overall/ultimate biliary cannulation success (allowing precut, if success occurred during that same procedure). Deep cannulation was defined as the tip of the catheter passing freely beyond the sphincter segment. Other cannulation maneuvers (e.g. wire-guided cannulation or temporary pancreatic stenting), without precut, were considered “conventional” techniques for our purposes.

ERCP case difficulty (complexity) was graded from 1 (standard) to 3 (tertiary ERCP), according to prior publication: grade 1 comprises ductography, brushing, biliary sphincterotomy, subcentimeter stone removal, subhilar stenting; whereas grade 3 includes therapeutics in surgically altered anatomy, manometry, ductoscopy, lithotripsy, intrahepatic stones, ampullectomy, pancreatic and pseudocyst therapy [20]. American Society of Anesthesiology (ASA) grade estimated comorbidities: I (healthy), II (mild systemic disease), III-V (severe systemic disease). Trainee involvement was graded by approximate percentage of time trainees handled the duodenoscope (0%, 1%-50%, 51%-99%, 100%), including cannulation and therapy, in any particular case. Sedation type included moderate/conscious sedation (i.e. without propofol or anesthesiologist monitoring), MAC (“monitored anesthesia care”, propofol-induced deep sedation), and general anesthesia.

Endoscopist-specific data were gathered at a baseline survey before Network participation. It had 6 categories for the endoscopist’s prior hands-on training volume: 0 (no formal training), 1–100, 101–150, 151–200, 201–250, >250 procedures. Years of prior ERCP practice was recorded. Lifetime volume (estimated cumulative number of prior ERCPs) and annual volume (estimated by number of ERCPs performed the preceding year) were surveyed.

In addition to the baseline survey data, 2 endoscopist-specific variables were created as surrogates of “efficiency” in standard cases: grade-1-case procedure time (median time from inserting to removing scope in grade-1-difficulty cases); grade-1-case fluoroscopy time (median fluoroscopy time in grade-1-difficulty cases).

Case-specific variables included: trainee involvement, difficulty, ASA, sedation, inpatient/outpatient status at time of ERCP, and indications (stone, imaging abnormality, chronic pain, abnormal liver enzymes, pancreatitis, tumor ablation, and post-surgical problems). Endoscopist-level variables included: country (United States, United Kingdom (UK), others), academic/community setting, experience in years, lifetime volume, annual volume, training volume, grade-1 procedure time, and grade-1 fluoroscopy time.

### Statistical analysis and power considerations

For most numerical variables (experience measures, grade-1-difficulty times), distributions were positively skewed, so they were split by median or quartile.

To account for inherent clustering (i.e., same endoscopist performing multiple procedures), a multilevel model with random intercepts was constructed. First, univariate multilevel logistic regression analyses were performed yielding adjusted cannulation success rates. Correlations between variables were evaluated using Spearman’ rank correlation coefficients. Second, variables with an adjusted univariate p-value < 0.2 were entered into multivariate multilevel logistic regression. A backward stepwise approach was used, and adjusted odds ratios (OR) and corresponding 95% confidence intervals were reported. All tests were 2-sided, and p values <0.05 were considered statistically significant (SAS v9.2 (SAS Institute Inc, Cary, NC)).

With 13,018 subjects, we had high (96%) power to detect very small (2%) cannulation rate differences, for case-specific variables. For endoscopist-level factors, power was lower but still reasonable: for 84 doctors (eg. stratified by median annual volume), differences in cannulation success rates of >11% (e.g. 85% vs. 96%) could be detected with 80% power, assuming an intraclass correlation coefficient of 0.4 within endoscopists.

## Results

In the Network, 13,018 ERCPs in native papillae were performed by 85 endoscopists. Qualifying endoscopists (contributing >30 cases), consecutively entered a median of 8.9 ERCP/week (range 5–27) over up to almost 3 years (median entry 80 days). Although consecutive entry was not able to be audited directly, each endoscopist’s *actual* entered case volume was higher than their respective *expected* case volume for this time frame (using annual volume at baseline survey), consistent with consecutive case entry. Conventional deep biliary cannulation success rate was 89.8% (ranging 63.9%-100% for different endoscopists). Precut sphincterotomy was performed in 876 (876/13018, 6.7%) ERCPs, and deep biliary cannulation was achieved in 745 (85.1%). Overall deep biliary cannulation success rate (including precut-assisted cases) was 95.6% (ranging 80.2%-100% for different endoscopists).

A conventional cannulation success rate threshold of 80% was exceeded by 73 (85.9%) endoscopists, and 90% by 42 (49.4%) endoscopists. An overall (including precuts) cannulation success rate threshold of 80% was exceeded by all endoscopists, and 90% by 71 (83.5%).

### Case-specific characteristics

Briefly, 6235 (47.9%) were grade 1 difficulty and 3746 (28.8%) were grade 3, 30.5% were classified as ASA III-V, and just over half (55.3%) had MAC or general anesthesia (Table 1). The pre-ERCP status of patients was evenly split between outpatient and inpatient. Trainees were involved in 4113 (31.6%) procedures. The most common indication (36.8%) was suspected stone.

**Table 1:** Summary of case-specific characteristics

**Variables**	**n (%)**
**Trainee involvement**	
0%	8905 (68.4)
1-50%	1794 (13.8)
51-99%	1389 (10.7)
100%	930 (7.1)
**ERCP difficulty**	
1	6235 (47.9)
2	3037 (23.3)
3	3746 (28.8)
**ASA grade**	
I	2480 (19.1)
II	6573 (50.5)
III	3509 (27.0)
IV	446 (3.4)
V	10 (0.1)
**Patient status at time of ERCP**	
Inpatient	6286 (48.3)
Outpatient	6732 (51.7)
**Sedation level**	
Moderate/conscious	5820 (44.7)
Propofol/MAC	5044 (38.8)
General anesthesia	2154 (16.6)
**Indications**	
Suspected or known stone	4791 (36.8)
Obstructive Jaundice	2381 (18.3)
Chronic pain	1984 (15.2)
Abnormal liver tests	1165 (9.0)
Chronic pancreatitis	1109 (8.5)
Biliary post-surgical problem (leak, stricture)	609 (4.7)
Clarification of biliary image findings	426 (3.3)
Pancreatitis (acute, active )	434 (3.3)
Tumor ablation	119 (0.9)

### Endoscopist-specific characteristics

Most endoscopists (71%) were from the US with 19% from the UK; other countries included Canada, Australia, Brazil, Norway, and Venezuela (Table 2). About half the endoscopists did not receive formal ERCP training, and there was a broad range of lifetime experience and volumes. Surrogates of ERCPist “efficiency” included median procedure and fluoroscopy times in grade-1-difficulty cases of 25 min and 3 min, respectively.

**Table 2:** Summary of the endoscopist-specific characteristics

**Variables**	**n/median (%/range)**
**Country setting-no. (%)**	
US	60 (70.6)
UK	16 (18.8)
Others	9 (10.6)
**Hospital setting-no. (%)**	
Academic	34 (44.7)
Community	42 (55.3)
**Volume in Training (%)**	
0	40 (47.1)
1-100	6 (7.1)
101-150	8 (9.4)
151-200	5 (5.9)
201-250	7 (8.2)
>250	19 (22.4)
**Years of ERCP (years)**	
Median (range, IQR)	12 (0–36, 6–20)
**Lifetime Volume**	
Median (range, IQR)	1200 (175–15000, 587–2500)
**Annual volume**	
Median (range, IQR)	150 (10–940, 90–239)
**Procedure time for grade 1 (minutes)**	
Median (range, IQR)	25 (10–48, 20–30)
**Fluoroscopy time for grade 1 (minutes)**	
Median (range, IQR)	3 (0.3-10.1, 1.9-4.6)

### Univariate multilevel logistic regression analysis

Five case-specific factors were significantly associated with *conventional* cannulation success, adjusted for doctor clustering (Table 3): trainees, difficulty, ASA, outpatient status, and indications. Of the endoscopist-specific factors, only country was significant.

**Table 3:** Univariate multilevel analysis of predicting factors for conventional deep biliary cannulation success rate

**Variables**	Adjusted^* ^**conventional cannulation success rate (%)**	**p value**
**Case-specific**		
** Trainee involvement**		<0.0001
0%	89.2	
1-50%	81.3	
51-99%	93.1	
100%	99.0	
**ERCP difficulty**		<0.0001
1	90.2	
2	89.5	
3	86.2	
**ASA grade**		<0.0001
I	92.1	
II	89.4	
III-V	87.7	
**Patient status at time of ERCP**		0.002
Inpatient	88.6	
Outpatient	90.5	
**Sedation level**		0.14
Moderate/conscious	88.8	
propofol /general	90.2	
**Indications**		<0.0001
Suspected or known stone	92.1	
Obstructive Jaundice	84.5	
Chronic pain	91.4	
Abnormal liver tests	90.3	
Chronic pancreatitis	88.9	
Biliary post-surgical problem	85.5	
Clarify biliary image findings	89.5	
Pancreatitis (acute, active )	86.6	
Tumor ablation	94.1	
**Endoscopist-specific**		
**Country setting**		0.04
US	90.5	
UK	86.4	
Other	87.8	
**Hospital setting**		0.51
Academic	90.2	
Community	89.2	
**Volume in Training**		0.31
0	89.3	
1-100	93.3	
101-150	89.1	
151-200	89.2	
201-250	84.9	
>250	90.4	
**Years of ERCP**		0.79
≤6	90.1	
7-12	89.2	
13-20	89.5	
>20	88.0	
**Lifetime volume**		0.81
≤587	89.3	
588-1200	89.8	
1201-2500	88.2	
>2500	89.8	
**Annual volume**		0.53
≤90	87.9	
91-150	88.4	
151-239	90.1	
>239	90.6	
**Procedure time for grade 1**		0.86
≤25	89.6	
>25	89.4	
**Fluoroscopy time for grade 1**		
≤3	90.0	0.43
>3	88.9	

^*^Adjusted success rates were obtained from multilevel logistic regression models that accounted for clustering of cases within endoscopists.

Similarly, 5 case-specific factors were significantly associated with *overall/ultimate* success (Table 4): trainees, difficulty, ASA, sedation type, and indications. Four endoscopist-specific factors were significant: country, annual volume, and practice “efficiency” surrogates (median procedure and fluoroscopy times in grade-1-difficulty cases).

**Table 4:** Univariate multilevel analysis of predicting factors for overall deep biliary cannulation success rate

**Variables**	Adjusted^* ^**overall cannulation success rate (%)**	**p value**
**Case-specific**		
**Trainee involvement**		<0.0001
0%	95.1	
1-50%	90.1	
51-99%	96.8	
100%	99.5	
**ERCP difficulty**		0.02
1	95.0	
2	95.7	
3	93.5	
**ASA grade**		<0.0001
I	96.7	
II	95.3	
III-V	93.0	
**Patient status at time of ERCP**		0.12
Inpatient	94.7	
Outpatient	95.4	
**Sedation level**		0.01
Deep (with propofol)/General	95.8	
Moderate/conscious	94.2	
**Indications**		<0.0001
Suspected or known stone	96.8	
Obstructive Jaundice	91.9	
Chronic pain	96.3	
Abnormal liver tests	95.1	
Chronic pancreatitis	93.7	
Biliary post-surgical problem	94.3	
Clarify biliary image findings	94.4	
Pancreatitis (acute, active)	91.7	
Tumor ablation	96.5	
**Endoscopist-specific**		
**Country setting**		0.03
US	95.5	
UK	91.9	
Other	96.3	
**Hospital setting**		0.86
Academic	94.9	
Community	94.7	
**Volume in Training**		0.78
0	95.3	
1-100	96.1	
101-150	94.3	
151-200	94.7	
201-250	92.2	
>250	95.1	
**Years of ERCP**		0.74
≤6	95.0	
7-12	95.6	
13-20	95.2	
>20	93.9	
**Lifetime volume**		0.10
≤587	93.7	
588-1200	93.8	
1201-2500	94.7	
>2500	96.8	
**Annual volume**		0.01
≤90	92.2	
91-150	94.0	
151-239	95.3	
>239	97.1	
**Procedure time for grade 1**		0.04
≤25	95.8	
>25	93.7	
**Fluoroscopy time for grade 1**		0.02
≤3	95.9	
>3	93.7	

^*^Adjusted success rates were obtained from multilevel logistic regression models that accounted for clustering of cases within endoscopists.

As expected, lifetime volume was moderately correlated with annual volume (r = 0.44, p = 0.0001), and years performing ERCP (r = 0.60, p < 0.0001), respectively; correlation was weaker between years of experience and annual volume (r = -0.13; p = 0.29). The endoscopist’s median grade-1-difficulty fluoroscopy and procedure times were correlated with one another (r = 0.69, p < 0.0001).

### Multivariate multilevel logistic regression analysis

Because of the above inter-correlated variables, lifetime volume and procedure time were dropped for multivariate modeling. Outpatients (OR 1.21 vs inpatients) independently predicted *conventional* success, whereas high ERCP difficulty level (OR 0.59), higher comorbidity (ASA II: OR 0.81; III-V: OR 0.77), and non-stone indications (obstructive jaundice: OR 0.51, post-surgical problems (e.g. leaks, post-operative strictures): OR 0.51, active pancreatitis: OR 0.67) were independent predictors for lower success rates (Table 5). Relationships with trainee involvement were complex: a high degree of trainee involvement (hands-on >50% of the case) was associated with higher success (OR 1.58) than not having a trainee, whereas low levels of trainee involvement were associated with failure. Of note, none of the endoscopist-specific factors was significant.

**Table 5:** Multivariate multilevel logistic regression analysis of predicting factors for conventional deep biliary cannulation success rate

**Variables**	**OR (95%CI)**	**p value**
**Case-specific**		
**Trainee involvement**		<0.0001
0%	Reference	
1-50%	0.53 (0.44-0.65)	
51-99%	1.58 (1.21-2.06)	
100%	11.96 (6.59-21.71)	
**ERCP difficulty**		<0.0001
1	Reference	
2	1.03 (0.88-1.20)	
3	0.59 (0.48-0.72)	
**ASA grade**		0.03
I	Reference	
II	0.81 (0.67-0.97)	
III-V	0.77 (0.63-0.94)	
**Sedation level**		0.11
Deep (with propofol)/General	Reference	
Moderate/conscious	0.84 (0.68-1.04)	
**Patient status at time of ERCP**		0.01
Inpatient	Reference	
Outpatient	1.21 (1.05-1.38)	
**Indications**		<0.0001
Suspected or known stone	Reference	
Obstructive Jaundice	0.51 (0.44-0.60)	
Chronic pain	1.16 (0.89-1.50)	
Abnormal liver tests	0.80 (0.63-1.02)	
Chronic pancreatitis	0.93 (0.72-1.22)	
Biliary post-surgical problem	0.51 (0.39-0.67)	
Clarify biliary image findings	0.77 (0.55-1.10)	
Pancreatitis (acute, active)	0.67 (0.49-0.92)	
Tumor ablation	2.15 (0.96-4.81)	
**Endoscopist-specific**		
**Country setting**		0.22
US	Reference	
Other	0.77 (0.46-1.30)	
UK	0.73 (0.49-1.09)	

Similar factors were found to be independently associated with *overall/ultimate* success, including trainee involvement, comorbidity index, and certain indications (Table 6). However, moderate/conscious sedation (OR 0.67 [95%CI, 0.49-0.92] versus deeper anesthesia) predicted lower success as an additional factor, yet outpatient status (significant for conventional cannulation) was not predictive of this outcome. Finally, in contrast to the conventional success model, two endoscopist-specific factors were significant for this outcome: annual volume (>239: OR 2.79 [95%CI, 1.46-5.31]), and efficiency, as measured by median grade-1-difficulty fluoroscopy time (≤3 min: OR 1.72 [95%CI, 1.10-2.69]).

**Table 6:** Multivariate multilevel logistic regression analysis of predicting factors for overall deep biliary cannulation success rate

**Variables**	**OR (95%CI)**	**p value**
**Case-specific**		
**Trainee involvement**		<0.0001
0%	Reference	
1-50%	0.50 (0.38-0.66)	
51-99%	1.55 (1.05-2.27)	
100%	9.16 (4.18-20.05)	
**ERCP difficulty**		0.01
1	Reference	
2	1.26 (0.99-1.59)	
3	0.70 (0.51-0.97)	
**ASA grade**		<0.0001
I	Reference	
II	0.78 (0.59-1.02)	
III-V	0.52 (0.38-0.70)	
**Sedation level**		0.01
Deep (with propofol)/General	Reference	
Moderate/conscious	0.67 (0.49-0.92)	
**Patient status at time of ERCP**		0.43
Inpatient	Reference	
Outpatient	1.09 (0.88-1.35)	
**Indications**		<0.0001
Suspected or known stone	Reference	
Obstructive Jaundice	0.45 (0.35-0.57)	
Chronic pain	1.03 (0.63-1.70)	
Abnormal liver tests	0.71 (0.47-1.08)	
Chronic pancreatitis	0.63 (0.41-0.98)	
Biliary post-surgical problem	0.53 (0.35-0.79)	
Clarify biliary image findings	0.58 (0.35-0.96)	
Pancreatitis (acute, active )	0.46 (0.29-0.71)	
Tumor ablation	1.28 (0.38-4.34)	
**Endoscopist-specific**		
**Country setting**		0.40
US	Reference	
UK	0.71 (0.41-1.22)	
Other	0.78 (0.36-1.66)	
**Annual volume**		0.01
≤90	Reference	
91-150	1.28 (0.72-2.29)	
151-239	1.85 (0.95-3.60)	
>239	2.79 (1.46-5.31)	
**Fluoroscopy time for grade 1**		0.02
>3	Reference	
≤3	1.72 (1.10-2.69)	

## Discussion

Deep biliary cannulation success in native papillae is a widely accepted measure of competence in ERCP during training, and quality of an endoscopist in ERCP practice. Identifying predictors for successful biliary cannulation in native papilla, both at a case- and at an endoscopist-/team-level, have important implications in improving the quality of ERCP and patient care. Further multivariate analyses suggest that only case-specific factors are significantly associated with conventional native papilla biliary cannulation success, and that endoscopist- and institution-level factors may not be as important.

Pre-procedure evaluations considering complexity and indications are important, to weigh anticipated success rates into decision-making and consent. Prior studies correlating higher difficulty score and lower success were heterogeneous without sufficient adjustment for confounders; Verma *et al.* found no correlation between conventional cannulation success and procedure difficulty for trainees [21]. Our results supported a relationship, although the absolute differences seen were small. Overall “case” complexity and difficulty is determined by many factors (of which cannulation is just one), and so, does not necessarily correlate with “cannulation” difficulty. The negative randomized trials of ERCP in mild to moderate acute gallstone pancreatitis should already limit its use in active pancreatitis due to limited efficacy [22]; but pancreatitis also predicted lower success rates in our study, perhaps related to duodenal edema, providing more reason to avoid this context. Obstructive jaundice (mostly cancers) predicts lower success than in suspected stone cases; this is in keeping with a recent randomized trial advising against ERCP in obstructive jaundice from surgically resectable tumors mostly because of morbidity related to cannulation/stenting failures and rescue procedures (69%/83% success in drainage at ERCP in community/academic centers, respectively) [23]. Post-surgical biliary issues (e.g. leaks, strictures) also predict lower success; this has not been previously reported; anatomic distortion, edema, or need for atypical positioning (e.g. supine) because of surgical wounds may contribute.

Trainee involvement (modeled as yes or no) has been shown to increase post-ERCP pancreatitis [24]. The British Society of Gastroenterology (BSG) study reported trainees reduced cannulation success to 54% [12]. However, we explored the proportion of involvement, and found conventional and precut-assisted cannulation success rates decreased only with more casual trainee involvement. Trainees with brief involvement in a case may cause papilla edema, and lower the chance of the supervisor’s success. Lastly, confounding could exist if more skilled endoscopists allowed more trainee hands-on time.

In the present study, high ASA score was surprisingly another factor predicting lower cannulation success. Tenuous sedation (requiring a more hurried procedure) or patient positioning (e.g. due to obesity) may hinder cannulation in some way. Our results suggest that outpatient ERCP, adjusted for other factors, may have a higher success rate, even though adverse event rates may be comparable [25,26]. Previous reports have found higher technical success rates achieved under deep sedation and general anesthesia than moderate (“conscious”) sedation because of better patient tolerance and compliance [27,28]. However, our results showed that deeper sedation only predicted success if precut was allowed; this may be explained by an improved ability to use advanced rescue techniques with deeper sedation. Country predicted success in univariate analysis, but when corrected for differences in sedation use and other factors, it did not; the univariate association is likely confounded by international sedation practice differences.

ASGE, based on learning curves, recommends at least 180–200 cases (at least 50% therapeutic) to achieve competency in cannulation [9]. A recent study, however, found that at least 350 supervised procedures were needed for an 80% native papilla biliary cannulation success rate [21]. In the ERCP network, 69.5% endoscopists received <200 procedures during their training (54.2% less than 100), comparable to a recent survey: 60.4% responders completed <180 training cases [29]. Training volumes did not predict cannulation success in our cohort. However, high-volume-trained endoscopists (>500) were under-represented; higher-volume training might have impact. Another consideration is that endoscopists with <200 cases in training tended to have higher years performing ERCP (data not shown); as such, their learning curve may have already risen and plateaued in practice, minimizing the apparent impact of their lower volume training.

Many believe that after proper training, experience (volume, years) and annual volume contribute to outcomes, but consensus on relative importance of annual volume vs cumulative experience, and on recredentialing volume thresholds, is lacking. The British Joint Advisory Group (JAG) recommends that endoscopists should perform >75 ERCPs/year [30]. Both an American [11] and Austrian study showed that >50 ERCPs/year had higher cannulation success [31], yet community endoscopists (median 50 ERCPs/year) demonstrated no associations between success and volume or experience [8]. Weaknesses of the above mentioned studies include the lack of separating out cannulation success with and without precut, or native vs cut/stented papillae. Our data showed that higher annual volume (using quartiles) had a small but nonsignificant trend toward higher conventional success and a significant trend toward overall (precut-allowed) success (>239 ERCP/yr: OR 2.79). Perhaps some of the higher overall success of the more active endoscopist might be due to their ability to comfortably use a more advanced rescue technique like precut sphincterotomy. We explored other cutoffs for annual volume (data not shown), including 50 as others have suggested, and 100; neither was significant.

Fluoroscopy time can be influenced by several procedural factors, as well as endoscopist and X-ray technician experience, trainee involvement, and equipment quality [32-35]. Being a radiation-efficient endoscopist (averaging ≤ 3 min use in grade-1 cases) predicted overall success, suggesting quality in one aspect of practice might be associated with quality in another, which is a novel concept.

There are limitations with our study. First, as stated above, we have lower power to detect the effects of some doctor-level factors on biliary cannulation success due to the modest number of endoscopists. Second, the self-reported data could not be audited for accuracy; however the anonymous design should have reduced bias. Despite this, we acknowledge there could have been some selective reporting of more successful procedures; the number of procedures entered appeared to be similar to the number expected for each provider (based on *a priori* reported volumes), so we hope this bias was minimal. In addition, the generalizability of our conclusions may be limited by the fact that the volunteering endoscopists may not reflect average ERCP practice worldwide; however, this more pertains to the overall success rates, and less likely to affect the generalizability of the predictors themselves. Fortunately, the spectrum of training, volume, years in practice, practice settings, and success rates are comparable to that of other studies, and does not suggest a homogenous, highly skilled cohort of tertiary clinicians.

## Conclusions

In conclusion, our results based on this unique international dataset indicate that case-specific factors may have greater impact on the most important ERCP quality metric (biliary cannulation success), than endoscopist-specific ones. Annual volume and sedation practices may influence ultimate success when precut-assisted success is not considered a failure. With regard to endoscopist experience factors, annual volume appears to perhaps be more important than prior experience, and the ideal volume (>200/year) may be considerably higher than the 50/year previously published. Further study, with an even larger number of endoscopists, could further explore the minimum annual volume for maintenance of ERCP competence.

## Abbreviations

ERCP: Endoscopic retrograde cholangiopancreatography; ASA: American society of anesthesiology; OR: Odds ratio; CI: Confidence interval; MAC: Monitored anesthesia care; US: United States; UK: United Kingdom; BSG: British society of gastroenterology.

## Competing interests

PBC and JR have done consulting for Olympus, who sponsored the registry, but otherwise, there are no potential conflicts of interest.

## Authors’ contributions

CYP participated in study design, acquisition of data, statistical analysis, interpretation of data, manuscript draft, and manuscript revision. PJN obtained funding and was involved in the statistical analysis and interpretation of data. PBC participated in study design, acquisition of data, and critical revision of the manuscript. DTL was involved in study design and revision of the manuscript. JR was responsible for study design, acquisition of data, statistical analysis, and critical revision of the manuscript. All authors read and approved the final manuscript.

## Pre-publication history

The pre-publication history for this paper can be accessed here:

http://www.biomedcentral.com/1471-230X/13/147/prepub
